# Genetic Predisposition of Donors Affects the Allograft Outcome in Kidney Transplantation; Polymorphisms of Stromal-Derived Factor-1 and CXC Receptor 4

**DOI:** 10.1371/journal.pone.0016710

**Published:** 2011-02-03

**Authors:** Jung Pyo Lee, Jong Bin Bae, Seung Hee Yang, Ran-hui Cha, Eun Young Seong, Yang Jin Park, Jongwon Ha, Myoung Hee Park, Jin Ho Paik, Yon Su Kim

**Affiliations:** 1 Seoul National University Kidney Research Institute, Seoul, Korea; 2 Seoul National University College of Medicine, Seoul, Korea; 3 Department of Internal Medicine, Seoul National University College of Medicine, Seoul, Korea; 4 Department of Internal Medicine, Pusan National University School of Medicine, Busan, Korea; 5 Department of Surgery, Seoul National University College of Medicine, Seoul, Korea; 6 Department of Laboratory Medicine, Seoul National University College of Medicine and Clinical Research Institute, Seoul, Korea; 7 Department of Pathology, Seoul National University College of Medicine, Seoul, Korea; University of Nebraska Medical Center, United States of America

## Abstract

Genetic interaction between donor and recipient may dictate the impending responses after transplantation. In this study, we evaluated the role of the genetic predispositions of stromal-derived factor-1 (SDF1) [rs1801157 (G>A)] and CXC receptor 4 (CXCR4) [rs2228014 (C>T)] on renal allograft outcomes. A total of 335 pairs of recipients and donors were enrolled. Biopsy-proven acute rejection (BPAR) and long-term graft survival were traced. Despite similar allele frequencies between donors and recipients, minor allele of SDF1 rs1801157 (GA+AA) from donor, not from recipients, has a protective effect on the development of BPAR compared to wild type donor (GG) (*P* = 0.005). Adjustment for multiple covariates did not affect this result (odds ratio 0.39, 95% C.I 0.20–0.76, *P = *0.006). CXCR4 rs2228014 polymorphisms from donor or recipient did not affect the incidence of acute rejection. SDF1 was differentially expressed in renal tubular epithelium with acute rejection according to genetic variations of donor rs1801157 showing higher expressions in the grafts from GG donors. Contrary to the development of BPAR, the presence of minor allele rs1801157 A, especially homozygocity, predisposed poor graft survival (*P* = 0.001). This association was significant after adjusting for several risk factors (hazard ratio 3.01; 95% C.I = 1.19–7.60; *P* = 0.020). The allelic variation of recipients, however, was not associated with graft loss. A donor-derived genetic polymorphism of SDF1 has influenced the graft outcome. Thus, the genetic predisposition of donor should be carefully considered in transplantation.

## Introduction

A variety of recently discovered potent immunosuppressive agents have significantly improved short-term renal allograft survival in the last two decades. However, long-term allograft survival has not changed significantly. Although various immunological and non-immunological factors may affect the outcome of an allograft, the genetic interactions between donors and recipients may also be an important aspect for the impending responses after organ transplantation. Recently, we demonstrated genetic interactions of the CCR5 cytokine between donors and recipients and its effects on allograft rejection and allograft survival [1]. Although acute rejection (AR) and subsequent chronic inflammatory processes are initiated by the presentation of donor-antigens to recipient T cells, they are the ultimate manifestations of active interactions between grafts and recipients that mobilize various types of cells and humoral factors.

Stromal-derived factor-1 (SDF1) is the ligand for chemokine CXC receptor 4 (CXCR4). SDF1 is up-regulated in various organs that respond to tissue damage from irradiation or hypoxia [2]. SDF1 has a crucial role in stem cell mobilization, differentiation, and homing and also is a highly potent chemoattractant for monocytes and naive T cells [3], [4]. Genetic polymorphism of SDF1 has been reported to have an effect on the expression of SDF1 [3], [4] and affects patient survival in liver transplantation [5]. SDF1 expression is elevated in chronic renal allograft rejection [6]. However, the role of SDF1 expression controlled by genetic polymorphisms has not been investigated thoroughly in the transplantation field.

We assume that the graft outcome is dependent not only on the recipient's immune responses but also on the responses of the graft as an active participant. Therefore, we hypothesized that genetic variations in SDF1 and CXCR4 of donors as well as recipients might alter leukocyte trafficking in the short-term and the tissue repair process in the long-term, and would thereby be associated with an increased risk of AR and long-term graft survival. To evaluate this hypothesis, we analyzed the genetic variations of SDF1 and CXCR4 with regard to donor-recipient genetic interactions in kidney transplantation.

## Results

### Baseline characteristics and Genetic variations of SDF1-CXCR4

All variables except for the recipient's gender were not significantly different between the BPAR (+) and BPAR (−) groups (Table 1). Allele frequencies were deduced from the genotype distribution. Minor allele frequencies of SDF1 polymorphisms [rs1801157 (G>A)] and CXCR4 polymorphism [rs2228014 (C>T)] were 0.264, 0.113 in recipients, respectively, and 0.249, 0.099 in donors. There was no significant difference in allele frequencies between recipients and donors. Observed allele frequencies did not differ significantly from expected allele frequencies based on conformity with the Hardy-Weinberg equilibrium.

**Table 1:** Baseline characteristics by incident biopsy-proven acute rejection.

	Total	BPAR (−)	BPAR (+)	*P* value
n	335	274	61	
Recipients' gender (% male)	66.3	63.9	77.0	0.049
Recipients' age (years)	36.5±14.5	35.9±14.5	39.4±14.6	0.083
Donors' gender (% male)	51.9	50.7	49.3	0.347
Donors' age (years)	38.1±11.5	38.1±11.4	38.2±12.3	0.978
No. of HLA mismatch	2.7±1.5	2.6±1.6	2.9±1.4	0.198
Hypertension (%)				0.803
No	35.5	35.4	36.1	
ex-hypertension	24.5	25.2	21.3	
Current hypertension	40.0	39.4	42.6	
Diabetes mellitus (%)				0.661
No	86.6	87.2	83.6	
PTDM	72.	6.6	9.8	
original DM	6.3	6.2	6.6	
No. of transplantation (%)				0.894
1^st^	96.4	96.3	96.7	
2^nd^	3.3	3.3	3.3	
3^rd^	0.3	0.4	0	
Donor type (%)				0.817
Living related	69.0	69.7	65.6	
Living unrelated	20.9	20.4	23.0	
Deceased	10.1	9.9	2.1	

Data are presented as mean ± standard deviation or proportion.

The *t*-test was used for continuous variables and the chi-square test was used for categorical variables. BPAR, biopsy-proven acute rejection; PTDM, posttransplant diabetes mellitus; DM, diabetes mellitus.

### Genetic variations and the development of acute rejection

Among 335 recipients, 61 recipients (18.2%) experienced BPAR after renal transplantation. Table 2 presents the results of analyses for the association between genotypes of the SDF1 and CXCR4 polymorphism and risk for BPAR. Minor allele of SDF1 rs1801157 (GA+AA) from donor, not from recipients, has a protective effect on the development of BPAR compared to wild type donor (GG) [odds ratio (OR) 0.39, 95% confidence interval (CI) 0.21–0.76, *P* = 0.005] (Table 2). In the additive model of genotype analysis, the odds ratio of BPAR per copy of donor variant allele (A) was 0.43, which was significant (95% CI 0.24–0.75, *P* for trend  = 0.003). Adjustment for multiple covariates did not significantly affect this result (model 3, OR 0.39, 95% CI 0.20–0.76, *P = *0.006] (Table 3). Allelic variation of recipients, however, was not significantly associated with the occurrence of BPAR. CXCR4 rs2228014 polymorphisms from donor or recipient did not affect the incidence of acute rejection. (Table 2, 3) When we examined 57 acute cellular rejection pathologic findings according to donor SDF1 rs1801157 genetic variations (GG, n = 43; GA, n = 13; AA, n = 1), there were no significant differences between the polymorphism and severity of acute rejection (Table S1).

**Table 2:** Genetic distribution of recipient/donor SDF1 and CXCR4 Single Nucleotide Polymorphisms.

		Donor			*P* valuefor trend	Recipient			*P* valuefor trend
	Genotype	BPAR (−), n (%)	BPAR (+), n (%)	OR (95% CI)		BPAR (−), n (%)	BPAR (+), n (%)	OR (95% CI)	
SDF-1 rs1801157	GG	138 (54.8)	43 (75.4)	1.00 (reference)	0.003	144 (53.7)	33 (55.9)	1.00 (reference)	0.460
	GA	89 (35.3)	13 (22.8)	0.47 (0.24–0.92)		103 (38.4)	24 (40.7)	1.02 (0.57–1.82)	
	AA	25 (9.9)	1 (1.8)	0.13 (0.02–0.98)		21 (7.8)	2 (3.4)	0.42 (0.09–1.86)	
	GA+AA vs. GG	114 (45.2)	14 (24.6)	0.39 (0.21–0.76)	0.005	124 (46.3)	26 (44.1)	0.92 (0.52–1.61)	0.759
	Total	252 (100)	57(100)			268(100)	59 (100)		
CXCR4 rs2228014	CC	201 (58.0)	45 (80.4)	1.00 (reference)	0.975	201 (78.8)	45 (81.8)	1.00 (reference)	0.463
	CT	43 (32.4)	11 (19.6)	1.14 (0.55–2.39)		49 (19.2)	10 (18.2)	0.91(0.43–1.94)	
	TT	3 (9.6)	0 (0.0)	NA		5 (2.0)	0 (0.0)	NA	
	CT+TT vs. CC	46 (42.0)	11 (19.6)	1.07 (0.51–2.22)	0.860	54 (21.2)	10 (18.2)	0.83 (0.39–1.75)	0.619
	Total	247 (100)	56 (100)			255 (100)	55(100)		

BPAR, biopsy-proven acute rejection; OR, odds ratio; CI, confidence interval; N/A, modeling not completed because SNP frequency = 0% in BPAR (−) and/or BPAR (+).

**Table 3:** The independent risk factor to the incidence of acute rejection analyzed by multivariate logistic regression.

	Donor			Recipient		
	OR	95% CI	*P* value	OR	95% CI	*P* value
**SDF-1 rs1801157**	GA+AA vs. GG			GA+AA vs. GG		
Model 1^a^	0.40	0.21–0.78	0.007	0.88	0.50–1.57	0.666
Model 2^b^	0.40	0.21–0.77	0.006	0.88	0.50–1.57	0.674
Model 3^c^	0.39	0.20–0.76	0.006	0.88	0.49–1.57	0.657
**CXCR4 rs2228014**	CT+TT vs. CC			CT+TT vs. CC		
Model 1^a^	1.12	0.53–2.34	0.772	0.90	0.42–1.93	0.793
Model 2^b^	1.11	0.53–2.34	0.778	0.92	0.43–1.98	0.831
Model 3^c^	1.11	0.53–2.34	0.785	0.92	0.43–1.97	0.819

a, adjusted for recipient's age and recipient's gender;

b, adjusted for recipient's age, recipient's gender, number of HLA mismatching, donor type;

c, adjusted for recipient's age, recipient's gender, number of HLA mismatching, donor type, number of transplantation, hypertension, and diabetes mellitus.

OR, odds ratio; CI, confidence interval.

### SDF1 expression according to SDF1 rs1801157 polymorphism

**Figure 1:**
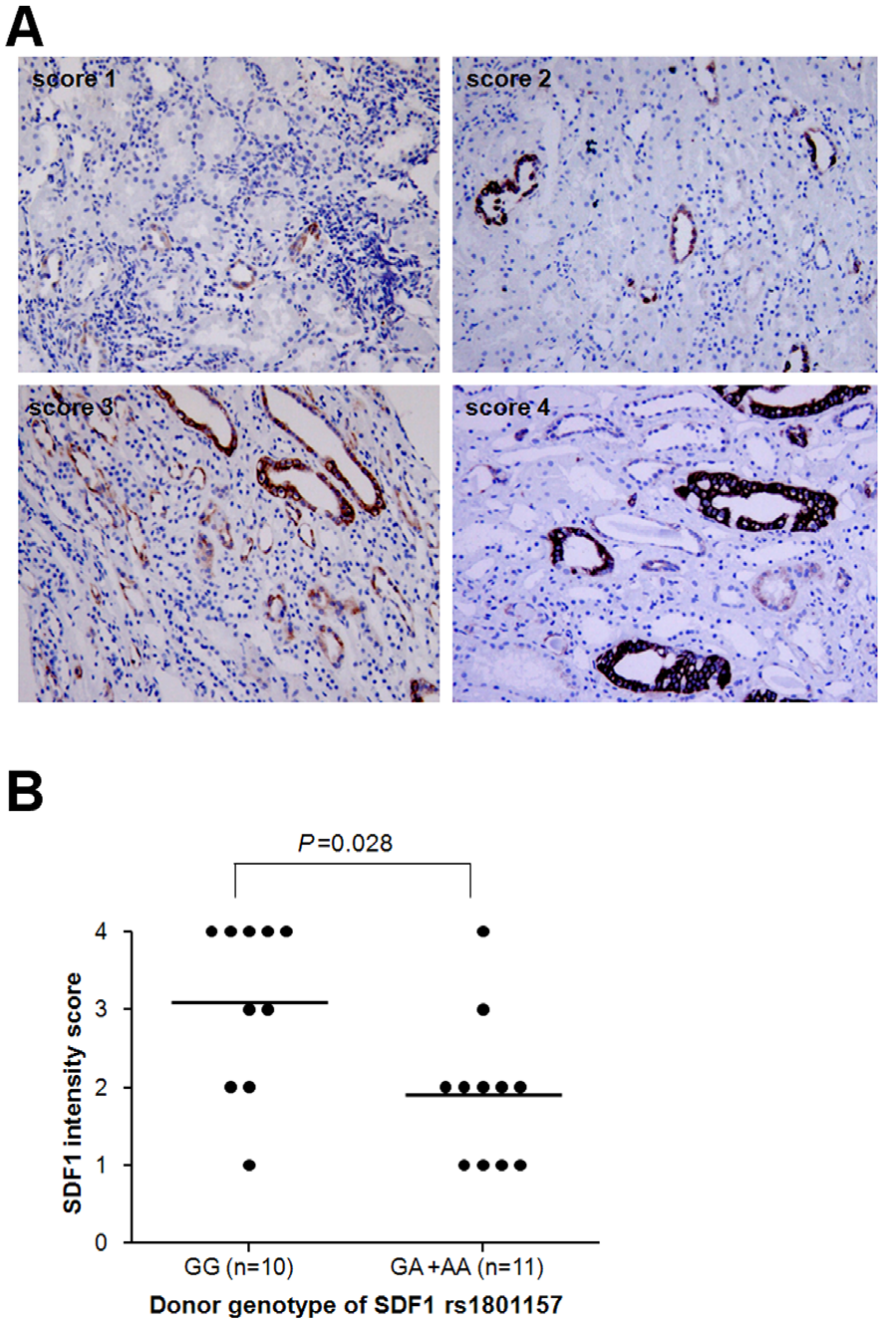
Semi-quantitative evaluation of the expression of SDF-1 in the kidney tubular epithelium proven acute cellular rejection. (A) Score 1, no staining or faint staining in a few tubules; score 2, weak staining; score 3, moderate staining; score 4, strong staining. (B) SDF-1 intensity scores according to donors' genetic variation of SDF-1 rs1801157, *P* value, Mann Whitney test.

The most prominent staining of SDF1 was detected in tubular epithelium. When we compared the expression of SDF1 according to donor SDF1 genotypes in 21 recipients who experienced BPAR, we found that SDF1 expression was significantly higher in the graft from donors with the wild-type genotype (GG) compared with the GA or AA genotype (mean staining score ± SE, 3.1±0.3 vs. 1.9±0.3, *P* = 0.028, Figure 1B). Recipients' polymorphisms did not affect SDF1 expression (data not shown).

### Long-term graft survival and SDF1 rs1801157 polymorphism

**Figure 2:**
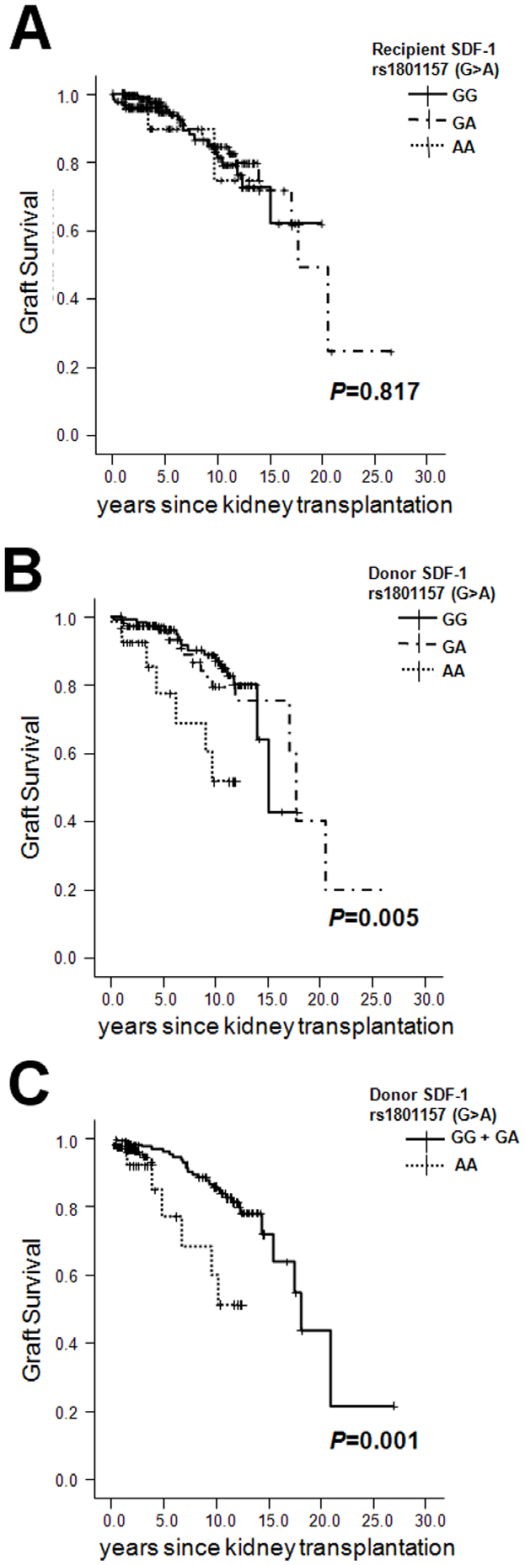
Graft survival according to SDF-1 rs1801157 (G>A) genotypes. (A) Recipients, and (B, C) donors. *P* value, log-rank test.

When we performed a survival analysis using the Kaplan-Meier method, recipients receiving the homozygous variant allele (AA) from donors showed poor graft survival compared to recipients who received kidneys with the wild-type (GG) or heterozygous genotype (GA) in the log-rank test, contrary to the association of BPAR and SDF1 polymorphism (Figure 2B and 2C, *P* = 0.005, 0.001, respectively). The median graft survival of recipients who had grafts from AA donors was significantly shorter than those of grafts from *GG* or *GA* donors (9.8 vs. 17.2 years). Again, recipients' polymorphisms did not affect graft survival.

**Figure 3:**
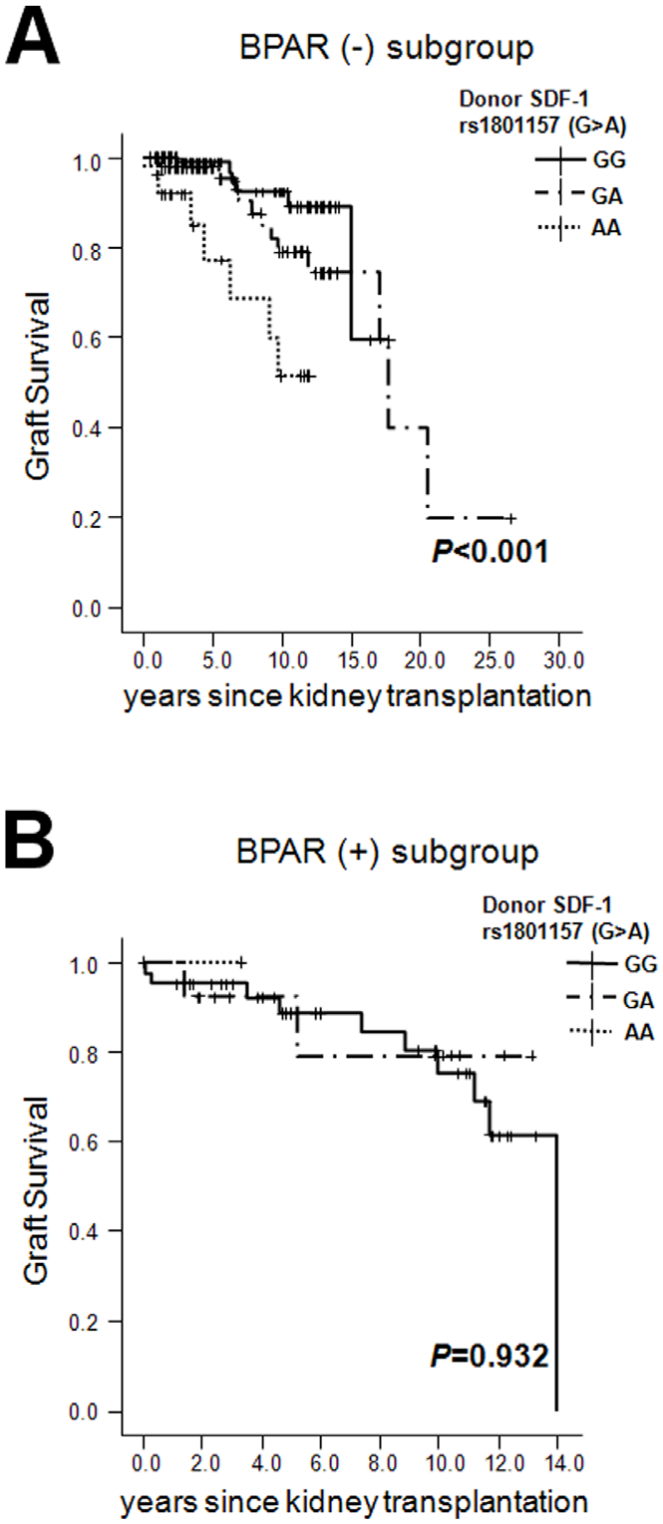
Graft survival according to donors' genetic variation of SDF-1 rs1801157 (G>A). (A) The biopsy-proven acute rejection (−) group, and (B) the biopsy-proven acute rejection (+) group. *P* value, log-rank test.

Next, we performed a subgroup analysis according to the presence of BPAR. In recipients who had not experienced BPAR, subjects with AA grafts had significantly poorer allograft survival (Figure 3A, *P*<0.001). In recipients experiencing BPAR, the number of recipients with *AA* grafts was too small. No one had lost an *AA* graft (Figure 3B, *P* value = 0.932).

Univariate and multivariate Cox regression analyses were performed after adjusting for several factors including BPAR. Unadjusted Cox regression analysis revealed that individuals carrying the AA genotype had an increased hazard ratio of graft loss (AA versus GG + GA genotype, hazard ratio = 3.63, 95% CI 1.57–8.39, *P* = 0.003). This association was statistically significant after adjusting for several risk factors (Model 5, hazard ratio  = 3.01; 95% CI 1.19–7.60, *P* value  = 0.020) (Table 4).

**Table 4:** Graft loss risk of recipients with donor SDF1 rs1801157 AA versus GG+GA by Cox regression analysis.

	Hazard ratio	95% confidence interval	*P* value
Model 1^a^	3.63	1.57–8.39	0.003
Model 2^b^	3.24	1.38–7.59	0.007
Model 3^c^	2.75	1.17–6.48	0.021
Model 4^d^	3.92	1.57–9.79	0.003
Modle 5^e^	3.01	1.19–7.60	0.020

a, unadjusted;

b, adjusted for recipient's age and gender;

c, adjusted for recipient's age, gender, number of HLA mismatching, donor type;

d, adjusted for recipient's age, gender, number of HLA mismatching, donor type, and biopsy-proven acute rejection.

e, adjusted for recipient's age, gender, number of HLA mismatching, donor type, biopsy-proven acute rejection, number of transplantation, diabetes mellitus and hypertension.

## Discussion

Our analysis identified a statistically significant association between the rs1801157 genetic variation at 3' UTR of SDF1 and graft outcome in terms of BPAR and graft loss after kidney transplantation. Specifically, the presence of the rs1801157 polymorphism variant allele of donors was associated with a significantly lower risk of BPAR, but it increased the risk of graft loss irrespective of BPAR.

Kidney tubular epithelial cells express cytokines, chemokines, or many of their receptors. They may play different roles in allograft rejection and chronic allograft dysfunction. Therefore, polymorphisms of cytokine and chemokine gene expression have been recognized as significant variables affecting allograft outcome. Recently, we reported that a CCR5 polymorphism in donors and recipients contribute together to provoke subsequent acute rejection in the early period and influence graft outcome in the later phase [1]. CXCR4 is the receptor of SDF1 and is expressed on several cells such as hemato/lymphopoietic cells, stem/progenitor cells, and several tumor cells [2]. SDF1 is up-regulated after ischemic injury to various organs [7]–[10] and leads to improved vasculogenesis and neovascularization through the recruitment of bone marrow-derived circulating endothelial progenitor cells to ischemic regions [11]. Besides these effects, it plays a crucial role in the retention of inflammatory cells [12]. Therefore, the SDF1/CXCR4 axis may be considered to be a potential target for therapeutic modifications. The SDF1/CXCR4 axis was suggested as an important regulator of allograft rejection and graft/patient survival. In liver recipients with acute and chronic allograft rejection, CXCR4+ T lymphocytes infiltrated SDF1 expressing bile ducts [13]. The SDF1 rs1801157 minor allele (A) of liver recipients was a significant risk factor for patient's survival, but did not affect rejection or graft survival [5]. In human heart transplantation, SDF1 was elevated and was involved in recruiting stem cells [14]. SDF1 positive cells infiltrate significantly more into the interstitium and arteries in chronic allograft nephropathy [8]. The SDF1 rs1801157 polymorphism came into the spotlight because the homozygous AA polymorphism delayed the onset of AIDS and prolonged survival of individuals infected with HIV-1 [15]. Afterward, this polymorphism has been investigated in many fields of study such as cancer and atherosclerosis [16]–[20]. CXCR7 has been found as a new receptor for SDF1. CXCR7 was expressed on both stem cells and inflammatory cells like CXCR4, although there has been some controversy until now [21]. Matthias AN et al. recently reported the role of chemokine receptor CXCR7 in renal allograft rejection [22]. They also revealed that SDF1 mRNA expression was significantly increased in acute allograft rejection. This finding is parallel with our present study.

This study showed that the presence of the rs1801157 variant allele (A) was associated with a significantly lower risk of BPAR, but it increased the risk of graft loss. It is unusual that one cytokine or chemokine performs in a different way in acute rejection than in chronic allograft nephropathy. In accordance with our results, IFN-γ prevented necrosis during a rejection process, but it potentiated chronic allograft dysfunction [23]. Yet, the role of SDF1 rs1801157 in 3′ UTR has not been well investigated in kidney transplantation. Although the SDF1 rs1801157 minor allele isoform (AA or GA) may significantly decrease plasma SDF1 level compared to the wild-type isoform (GG) [3], the relationship between the rs1801157 polymorphism and plasma SDF1 level or stem cell number is still controversial [4]. Although donor SDF1 polymorphisms could not alter plasma level after operation, intragraft SDF1 expression might be influenced [24], consequently leading to a change in cellular recruitment. If the rs1801157 minor allele (A) polymorphism decreased intragraft SDF1 expression, there would be less inflammation in the early period, and a decrease in acute rejection. At the later phase, however, it would reduce tissue repair and make chronic allograft nephropathy more severe. Although the number of biopsies performed with subsequent immunochemical staining per genotype in our study was small, the pattern in acute rejection is in agreement with the above-mentioned hypothesis.

Our results are informative, but this study has some limitations. First, the number of recipients transplanted from AA donor, who had a significant low graft survival, was small. There may be a likelihood of type 1 error. Study dealing with more large number of population is necessary to confirm our finding that polymorphism of SDF1 affect graft survival. Second, the protocol biopsy and works for the functional relationship of the SDF1 rs1801157 sequence variation of donor with BPAR or renal graft loss will be necessary.

In conclusion, a donor-derived, not recipient-derived, genetic SDF1 polymorphism has different effects on graft outcome. Thus, the genetic influence from donors should be carefully considered for the proper management of allografts after kidney transplantation.

## Materials and Methods

### Patients

A total of 335 pairs of Korean recipients and donors who were followed-up for at least 1 year were recruited for this study. They had received kidney transplants at Seoul National University Hospital between 1982 and 2008. We collected whole blood samples from the patients and their donors as follows: 307 samples from donors and recipients; 22 from recipients only; 6 from a donor only. The research protocol used for this study was approved by the Internal Review Board of Seoul National University Hospital (IRB No.H-0911-011-299). Written informed consents have been obtained and all clinical investigations have been conducted according to the principles expressed in the Declaration of Helsinki. We conducted medical record reviews of recipients based on the electronic medical record system. Clinical parameters that could have influenced graft outcome were collected, i.e. recipient's gender and age at transplantation, history of hypertension, diabetes mellitus, and donor type. The primary outcome of this study was biopsy-proven acute rejection (BPAR) within 1 year of the transplant. The secondary outcome was graft loss which was defined as graft dysfunction that necessitated new renal replacement therapy after transplantation.

### Genotyping

DNA was extracted from whole blood, and genotyping for SDF1 [rs1801157 (G>A)] and CXCR4 [rs2228014 (C>T)] was carried out by the TaqMan® method (Applied Biosystems, 7900HT Fast real time PCR system, USA). Primers used for SDF1 and CXCR4 were as follows: SDF1 rs1801157 5′-GTGAAGGCTTCTCTCTGTGGGA-3′ and 5′-TCTGCCCTGGCCTCACAC-3′; CXCR4 rs2228014 5′-CAACCTCTACAGCAGTGTCCTCAT-3′ and 5′-CAGCTTCCTTGGCCTCTGAC-3′.

A different fluorescence label [6-carboxyfluorescein (FAM) for mutant and 6-carboxy-4,7,2′,7′-tetrachlorofluorescein (TET) for wild type] was used to label the 5′ segment of allelic probes. Probe sequences as follows: SDF1 rs1801157 5′ (FAM)-CATGGGAGCC**G**GGTCTGCC-3′ (TAMRA) and 5′ (TET)-CATGGGAGCC**A**GGTCTGCCTCTT-3′ (TAMRA); CXCR4 rs2228014 5′ (FAM)-CGCTACCTGGCCAT**C**GTCCA-3′ (TAMRA) and 5′ (TET)-CTGGCCAT**T**GTCCACGCCAC-3′ (TAMRA). Reaction mixtures consisted of a 1.0 µL 10× AmpliTaq buffer, 1.0 µL deoxynucleotide triphosphates (2.5 mM each), 0.2 µL forward primer (20 pmol/µL), 0.2 µL reverse primer (20 pmol/µL), 1.0 µL genomic DNA (50 ng/µL) and 0.15 µL iMax II Taq polymerase. PCR reactions were carried out under the following conditions: 5 min 94°C (one cycle); 30 s 94°C; 30 s 56°C (35 cycles); 50 s 72°C, 7 min 72°C (one cycle). PCR products were analysed on 2% agarose gels. Confirmation of genotypes was done by repeated PCR and DNA sequencing using the ABI Prism BigDye Terminator Kit (Applied Biosystems, Foster City, CA, USA) of 10% of the study population samples.

### Tissue immunohistochemistry staining and analysis

For the immunohistochemistry study, paraffin embedded graft blocks of recipients diagnosed as BPAR were collected and cut into 4 µm slices. Xylene and ethanol were used for deparaffinization and hydration. Endogenous streptavidin activity was blocked by 3% hydrogen peroxide (H_2_O_2_). To examine the expression of human SDF1, deparaffinized sections were stained with 1∶200 diluted anti-SDF1 antibody (R&D Systems, Minneapolis, MN) and incubated at 4°C overnight. Sections were then incubated with biotin-conjugated anti-mouse IgG biotinylated secondary antibody (Dako, Carpintería, CA). Streptavidin and 3, 3′-diaminobenzidine tetrahydrochloride (Sigma–Aldridge, St. Louis, MO) were used for immunohistochemical detection. Sections were then counterstained with Mayer's hematoxylin and examined by light microscopy. The SDF1 score was graded in a blinded fashion by a renal pathologist. The score was expressed semi-quantitatively as 1 to 4 as follows. Score 1 indicated basically no staining or faint staining in a few tubules, score 2 indicated weak staining, score 3 corresponded to moderate staining, and score 4 signified strong staining (Figure 1A).

### Statistical analysis

We used SPSS for Windows package 12.0K (SPSS Inc., Chicago, IL) for all analyses and calculations. Student's *t*-test was used for continuous variables, and they were presented as mean ± SD. The chi-square test was used for categorical variables. Graft survival was analysed using the Kaplan–Meier method, and comparison among groups was performed by the log-rank test. We performed multivariate analysis using a binary logistic regression test for risk of BPAR and the Cox regression test for risk of graft loss, respectively (backward stepwise method). Values of *P<*0.05 were considered as statistically significant.

## Supporting Information

Table S1 **Association between donor SDF-1 rs1801157 (G>A) genetic variations and severity of acute cellular rejection.**
(DOC)
