# Supplementary material for: Genetic Predisposition of Donors Affects the Allograft Outcome in Kidney Transplantation; Polymorphisms of Stromal-Derived Factor-1 and CXC Receptor 4
Source: PLoS One. 2011 Feb 3;6(2):e16710. doi: 10.1371/journal.pone.0016710 (PMC3033398; doi:10.1371/journal.pone.0016710)
Supplement: Table S1 — Association between donor SDF-1 rs1801157 (G>A) genetic variations and severity of acute cellular rejection. (DOC) [file pone.0016710.s001.doc]

**Table S1. Association between donor SDF-1 rs1801157 (G>A) genetic variations and severity of acute cellular rejection.**

|  | GG (n=43) | GA+AA (n=14) | *P* value |
| --- | --- | --- | --- |
| Serum creatinine (mg/dL) at graft biopsy | 3.0 ± 2.2 | 2.0 ± 0.7 | 0.003 |
| Months to biopsy | 0.8 ± 1.2 | 0.5 ± 0.3 | 0.312 |
| Banff classification 2009 (%)^*^ |  |  | 0.458 |
| I A | 34.9 | 28.6 |  |
| I B | 4.7 | 7.2 |  |
| II A | 53.4 | 50.0 |  |
| II B | 4.7 | 14.2 |  |
| III | 2.3 | 0.0 |  |
| Tubulitis^†^ | 1.7 ± 0.9 | 1.9 ± 1.0 | 0.921 |
| Intimal arteritis^†^ | 0.7 ± 0.6 | 0.9 ± 0.7 | 0.496 |
| Interstitial infiltration^†^ | 2.1 ± 0.8 | 2.4 ± 0.5 | 0.321 |

*graded in accordance with Banff classification 2009: IA, Cases with significant interstitial infiltration (>25% of parenchyma affected, i2 or i3) and foci of moderate tubulitis (t2); IB. Cases with significant interstitial infiltration (>25% of parenchyma affected, i2 or i3) and foci of severe tubulitis (t3); IIA, Cases with mild to moderate intimal arteritis (v1); IIB, Cases with severe intimal arteritis comprising >25% of the luminal area (v2); III, Cases with ‘transmural’ arteritis and/or arterial fibrinoid change and necrosis of medial smooth muscle cells with accompanying lymphocytic inflammation (v3);

†graded semi-quantitatively on a scale of 0–3.
